# *Staphylococcus aureus* in Polymicrobial Skinand Soft Tissue Infections: Impact of Inter-Species Interactionsin Disease Outcome

**DOI:** 10.3390/antibiotics12071164

**Published:** 2023-07-08

**Authors:** Florencia Mariani, Estela Maria Galvan

**Affiliations:** 1 Laboratorio de Patogénesis Bacteriana, Departamento de Investigaciones Bioquímicas y Farmacéuticas, Centro de Estudios Biomédicos, Biotecnológicos, Ambientales y Diagnóstico (CEBBAD), Universidad Maimónides, Hidalgo 775, Buenos Aires C1405, Argentina; mariani.florencia@maimonides.edu; 2 Consejo Nacional de Investigaciones Científicas y Técnicas (CONICET), Buenos Aires A4400, Argentina

**Keywords:** *Staphylococcus aureus*, skin and soft tissue infection, polymicrobial infection, interspecies interactions

## Abstract

Polymicrobial biofilms provide a complex environment where co-infecting microorganisms can behave antagonistically, additively, or synergistically to alter the disease outcome compared to monomicrobial infections. *Staphylococcus aureus* skin and soft tissue infections (Sa-SSTIs) are frequently reported in healthcare and community settings, and they can also involve other bacterial and fungal microorganisms. This polymicrobial aetiology is usually found in chronic wounds, such as diabetic foot ulcers, pressure ulcers, and burn wounds, where the establishment of multi-species biofilms in chronic wounds has been extensively described. This review article explores the recent updates on the microorganisms commonly found together with *S. aureus* in SSTIs, such as *Pseudomonas aeruginosa*, *Escherichia coli*, *Enterococcus* spp., *Acinetobacter baumannii*, and *Candida albicans*, among others. The molecular mechanisms behind these polymicrobial interactions in the context of infected wounds and their impact on pathogenesis and antimicrobial susceptibility are also revised.

## 1. Introduction

Skin and soft tissue infections (SSTIs) comprise a group of infections that affect the skin and underlying subcutaneous tissue, fascia, or muscle. These infections can vary in severity, ranging from superficial infections of mild to moderate severity to deeper necrotizing infections [1,2]. SSTIs have significant global impact, increasing hospitalizations, length of stay, and mortality [3,4,5].

Several classifications can be adopted for SSTIs, depending on specific variables such as anatomical localization, etiological agent(s), skin extension, progression rate, clinical presentation, and severity [6,7,8,9]. The Infectious Diseases Society of America (IDSA) classification is based on three different distinctions: (i) skin extension, uncomplicated, typically superficial infections, and complicated infection, basing the latter definition for those reaching deep structures of the skin; (ii) rate of progression, acute wound infections (traumatic, bite-related, postoperative) and chronic wound infections (diabetic foot infections, venous stasis ulcers, pressure sores); (iii) tissue necrosis, necrotizing (fascitis, myonecrosis, gangrena) and non-necrotizing infections [7]. There is one last criterion that allows the differentiation of SSTIs as monomicrobial and polymicrobial [3]. Especially those infections with a long lasting or chronic course can be sustained by multiple microbial species [10].

All these classifications include patients with the following clinical entities: (i) cellulitis/erysipelas, defined as a skin infection characterised by spreading areas of redness, edema, or induration; (ii) wound infection, characterised by purulent drainage from a wound with surrounding redness, edema, or induration; and (iii) major cutaneous abscess characterised by a collection of pus within the dermis or deeper that is accompanied by redness, edema, or induration [3].

*Staphylococcus aureus*, an opportunistic Gram-positive pathogen, is a common cause of SSTIs, ranging from the benign (e.g., impetigo and uncomplicated cellulitis) to the immediately life-threatening [3,11]. Among *S. aureus* strains, methicillin-resistant *S. aureus* (MRSA) isolates are of particular concern because they can also exhibit concomitant resistance to many commonly used antibiotics. The specific multidrug-resistant pattern of MRSA can vary depending of the geographic location and includes resistance to macrolides (erythromycin and clarithromycin), lincosamides (clindamycin), aminoglycosides (gentamicin), tetracyclines (tetracycline and doxycycline), and fluoroquinolones (ciprofloxacin) [12,13,14].

*S. aureus* expresses several factors that facilitate skin colonization and infection. These include various toxins and immune evasion factors, and a large array of protein and non-protein factors that enable host colonisation during infection [15,16]. *S. aureus* avoids being eliminated by neutrophils on many levels that include: (i) the inhibition of neutrophil extravasation from the bloodstream into the tissues, neutrophil activation, and chemotaxis, (ii) inhibition of phagocytosis by aggregation, protective surface structures, and biofilm formation, (iii) inhibition of opsonisation, (iv) inhibition of neutrophil killing mechanisms, and (v) direct elimination of neutrophils by cytolytic toxins or triggering of apoptosis [16,17].

Furthermore, biofilm formation has been postulated as a common behaviour of *S. aureus* isolates from skin infections [18,19,20,21]. Biofilms pose a significant challenge in the treatment of these infections due to their unique characteristics and the protective environment they create. In this regard, biofilms show increased resistance to host immunity and increased tolerance to antibiotics compared to their planktonic counterparts [22,23].

Polymicrobial SSTIs involving *S. aureus* have been reported (Sa-SSTIs) [10,11,24,25,26]. Most mixed-species SSTIs are associated with chronicinfections such as diabetic foot infections (DFIs), pressure ulcers infection, and burn infection, among others [10]. These chronic wounds can commonly become infected with polymicrobial biofilms containing bacterial and fungal microorganisms [27,28]. Mixed biofilm communities provide a complex environment in which a variety of interactions may occur, ranging from cooperative interactions to antagonism [29,30]. The polymicrobial interactions in wounds may help the partner species to establish and infect the tissues. It has been reported that the high polymicrobial load in wounds delays the wound closure and favours the emergence of antibiotic-resistant strains compared to the single-species biofilms [31,32]. Understanding the microbial species involved, predisposing factors of the disease progression, and the polymicrobial interaction between microorganisms is essential for diagnosing and developing treatment strategies.

This review article explores the recent updates on the microorganisms commonly found together with *S. aureus* in SSTIs, such as *Pseudomonas aeruginosa*, *Escherichia coli*, *Enterococcus* spp., *Acinetobacter baumannii*, and *Candida albicans*. The molecular mechanisms behind these polymicrobial interactions and their impact on pathogenesis and antimicrobial susceptibility are also revised.

## 2. Occurrence of PolymicrobialSSTI Associated with *S. aureus* (Sa-SSTIs)

Infections with a long-lasting or chronic course are usually sustained by multiple microbial aetiologies [30,33,34,35]. In this regard, polymicrobial SSTIs are usually observed for diabetic foot ulcers, pressure ulcers, and burn wounds [3,10]. Microbiological assessment of polymicrobial SSTIs, performed by standard culturing techniques or molecular methods, can be challenging [3,36,37]. Culture-dependent techniques are biasedtoward those microorganisms that develop well under laboratory conditions, and might inadequately represent fungal and bacterial communities in chronic wounds [20,38]. On the other hand, culture-independent, amplicon-based sequencing methods (i.e., bacterial and fungal 16S rRNA gene sequencing) have the major limitation of failing to distinguish individual species [21,39]. Recently, exhaustive strain-level classification of microbial communities has been achieved by shotgun metagenomic sequencing [40]. Consequently, a combinationof metagenomic approach and culturing methods seems to be more adequate to identify the complex microbial communities formed in chronic wounds [41].

The microbiology of SSTIs shows that *S. aureus* is a frequent aetiology, with a high incidence of MRSA [2,8]. Cumulative data indicate that up to 70% of Sa-SSTIs are polymicrobial [2,10,25]. As mentioned early, mixed-species SSTIs are usually associated with chronic infections such as diabetic foot infections (DFIs), pressure ulcers infection, and burn infections [3].

### 2.1. Diabetic Foot Infections (DFIs)

Diabetic foot ulcers are a common complication of diabetic patients affecting their lower extremities; these wounds occur due to a combination of factors such as reduced blood flow and nerve damage (neuropathy) [42,43]. Although usually initially characterised as acute wounds, their inability to progress through the healing stages converts them into chronic wounds [11].

Diabetic foot ulcers are highly susceptible to infections due to several reasons: (i) diabetes can cause peripheral neuropathy, which damages the nerves in the feet, making it difficult for the patient to notice a foot ulcer infection developing; (ii) compromised blood circulation that impairs the immune cells to reach the wound efficiently; and (iii) prolonged healing due to the underlying complications mentioned before, which provides an extended window of opportunity for bacteria to multiply and establish an infection. Once an ulcer becomes infected, the bacteria can spread through the tissues, leading to cellulitis, abscess formation, osteomyelitis (infection of the bone), or systemic infection if left untreated. In severe cases, the infection can progress to a point where amputation becomes necessary [44].

Chronic diabetic foot ulcers usually become infected with bacterial biofilms, which constitute a significant factor contributing to the severity and delayed healing of diabetic foot infections (DFIs) [20]. Diabetic foot ulcers are typically colonised with skin commensal bacteria establishing biofilms that increase their microbial diversity over time and with progression of the ulcer [21,45]. Some common microorganisms associated with DFIs are *Staphylococcus* spp., *Corynebacterium* spp., and *P. aeruginosa* [26,46]; however, these infections involve a great diversity of microbes. The microorganisms reported to co-exist with *S. aureus* in polymicrobial DFIs are mainly gram negative bacteria: *P. aeruginosa*, *Acinetobacter* spp., *Escherichia coli*, *Enterobacter* spp., *Citrobacter* spp., *Proteus* spp., *Klebsiella* spp. In addition, Gram-positive *Enterococcus* spp. have also been reported to co-occur with *S. aureus* in DFIs (Table 1) [10,27].

### 2.2. Pressure Ulcer Infections

Pressure ulcers (PUs) are injuries to the skin and underlying tissue resulting from ischemia caused by prolonged pressure on the skin [28]. PUs can affect any part of the body that is put under pressure. They are most common on bony parts of the body, such as the heels, elbows, hips, and base of the spine [53]. PUs are a significant health problem worldwide that commonly occurs among inpatients and elderly people with physical-motor limitations. The overall prevalence of pressure ulcers in hospitalised patients has been estimated to range from 5% to 15% but may be significantly higher in intensive care units and certain long-term care settings [54,55].

PUs are typically categorised into stages based on their severity: (i) Stage 1, the skin is intact, but there may be non-blanchable erythema; (ii) Stage 2, partial-thickness skin loss with exposed dermis; (iii) Stage 3, full-thickness skin loss: (iv) Stage 4, full-thickness skinand tissue loss; (v) unstageable pressure injury, obscured full-thickness skinand tissue loss; (vi) deep tissue pressure injury, persistent nonblanchable deep red, maroon, or purple discoloration [54,55].

These wounds are frequently exacerbated by the presence of bacteria and advanced stages of PUs are described to be polymicrobial and linked with biofilm-associated infections [28,29]. The most common organisms identified in PUs are *S. aureus*, *Proteus mirabilis*, *P. aeruginosa*, and *E. faecalis* [53]. In chronically infected PUs, *S. aureus* has been found together with *P. aeruginosa*, *E. coli*, *P. mirabilis*, *Enterobacter cloacae*, and *E. faecalis* (Table 1) [29,47,48].

### 2.3. Burn Wound Infections

Burn wounds refer to injuries that result from exposure to heat, chemicals, electricity, or radiation, and they are considered a public health issue all over the world, especially in low- or middle-income countries [56,57]. Burn wounds can vary in severity and are typically classified, based on the depth and extent of tissue damage, as follows: (i) first-degree burns, superficial burns, called erythema, that only affect the epidermis; (ii) second-degree burns, partial-thickness superficial burns where the epidermis and the dermis are damaged; (iii) third-degree burns, full-thickness deep burns that affect all layers of the skin, including the subcutaneous tissue and the muscle; (iv) fourth-degree burns, full-thickness burns including deeper lying tissues such as muscles, tendons, or bones [58].

Burn wounds are particularly susceptible to infections because the damaged skin provides an entry point for microbes, including bacteria and fungi. Microbial infections in burn wound patients are difficult to control; moreover, biofilm formation in burns is a major concern [49,59]. Some of the bacteria commonly found in chronic burn wound infections are *P. aeruginosa*, *S. aureus*, *Streptococcus* spp., *Klebsiella* spp., *Enterococcus* spp., and *E. coli*. In addition, the most prevalent fungi are *Aspergillus niger* and *Candida* spp. [60]. In particular, chronic burn wounds co-infected by *S. aureus/P. aeruginosa* and *S. aureus/C. albicans* have been widely reported (Table 1) [49,50,51,52].

## 3. Implications of Polymicrobial Interactionson Infection Outcome

Polymicrobial infections, which are being recognised with increasing frequency, can occur in various parts of the body including the oral cavity, respiratory tract, urinary tract, skin, and wounds [30,61,62]. The presence of multiple microbial species in a polymicrobial infection can lead to several challenges in diagnosis, treatment, and management [61,63,64]. This is partly because infectious polymicrobial communities are often found to be more resistant to antibiotics than their mono-culture counterparts [65,66].

A polymicrobial biofilm is a complex community of microorganisms (fungi, bacteria, and viruses) that adhere to a biotic or abiotic surface, and it is embedded in a self- and/or host-derived hydrated matrix, often consisting of polysaccharides, proteins, and extracellular DNA [30,67]. Biofilm formation involves a series of steps: aggregation or attachment of cells to a surface, growth of the cells into a sessile biofilm colony, and detachment of the cells from the colony into the surrounding medium [22,68]. Because of the large variety and concentration of microbes present in polymicrobial biofilms, each of these stages can be shaped by species-specific physical and chemical interactions, ranging from cooperative relationships to microbial competition [34,69].

### 3.1. Beneficial Interactions

In polymicrobial biofilms, the synergism and cooperation between microbial species are important to keep the coexistence of different microorganism, outcompeting possible mutual antagonistic effects [70,71].

A behaviour that helps to promote multispecies coexistence within a biofilm occurs when microbes initiate cohesion and coaggregation by producing several adhesion molecules that induce intercellular interactions. Coaggregation has been very well studied in the oral biofilm–dental plaque, and it can involve fimbriae, other surface proteins with adhesive properties, and extracellular polysaccharides; for example, the short fimbriae of *Porphyromonasgingivalis* play a role in coadhesion with *Streptococcus gordonii* [72,73].

Another important cooperative strategy is related to metabolic interactions, such as cross-feeding. This occurs when different strains have access to distinctive nutrient substrates, and the product of one strain’s metabolism can be utilised in the nutrition of another. An example is that of *Aggregatibacter actinomycetemcomitans* and *Streptococcus gordonii*, bacteria isolated from the human oral cavity. It has been shown that *Streptococcus gordonii* can secrete lactate as a metabolic byproduct, and this lactate is used as a preferred carbon source by *Aggregatibacter actinomycetemcomitans*, favouring its growth [74]. Cross-feeding is beneficial because it gives single or multispecies biofilm systems higher metabolic efficiency that can better support the growth of the microorganisms [71,75].

Quorum sensing, a type of cell signalling related to the ability to detect and respond to cell population density by gene regulation, is important in biofilm formation and interspecies communication. Quorum sensing acts through small diffusible signal molecules (autoinducers) that have been implicated in interspecies cooperation [71]. In this regard, Autoinducer 2 (AI-2) produced by *Enterococcus faecalis* promotes collective behaviours of *Escherichia coli* at lower cell densities, enhancing autoaggregation of *E. coli* but also leading to chemotaxis-dependent coaggregation between the two species [76].

In addition, within biofilms, different species of bacteria can use horizontal gene transfer to exchange antibiotic resistant genes, helping the entire community survive antibiotic exposure. The mechanisms of horizontal gene transfer include conjugation, transformation, transduction, membrane vesicles, and gene transfer agents [77,78,79]. It has been widely reported that horizontal gene transfer allows microbes to acquire new sources of antibiotic resistant genes [80]. For example, one study described that a plasmid harboring a carbapenemase resistance gene (*bla*_NDM-1_) can be transferred from *E. coli* to either *P. aeruginosa* or *Acinetobacter baumannii* via conjugation within dual-species biofilms [81].

Finally, microorganisms in polymicrobial biofilms may benefit each other by secreting certain beneficial molecules, such as enzymes that inactivate detrimental agents. In this context, studies of polymicrobial biofilms related to otitis media evidenced that beta-lactamase production by *Moraxella catarrhalis* provides passive protection to *Streptococcus pneumoniae* from beta-lactam antibiotic killing [82].

### 3.2. Competitive Interactions

Bacteria in mixed-species biofilms have to coexist and compete for limited space and nutrients. Competition between species appears to define the interactions that predominate in microbial communities [83]. Competition is categorized into two modes, exploitative and interference. Exploitative competition refers to indirect interactions between organisms, by which one organism prevents access to and/or limits the use of resources by another organism whereas interference competition is related to the production of antagonistic factors to impede competitors [84,85,86].

In biofilms, bacteria live under severe environmental conditions, characterized by low nutrient concentrations and low rates of gas renewal or exchanges [68]. Due to the requirements for limited nutrients, different bacterial species compete for nutrients to survive. Competition for iron has been widely observed and is related to the production of iron-chelating molecules (siderophores) by microorganisms [69]. For example, iron competition has been postulated to modulate bacterial composition of dual-species biofilms formed by uropathogenic *Klebsiella pneumoniae* and *E. coli* strains, promoting *K. pneumoniae* growth to the detriment of *E. coli* [87]. Moreover, oxygen competition has been described between aerobic microorganisms growing in polymicrobial biofilm pellicles at the air liquid interface. For instance, the facultative aerobe *Pseudoxanthomonas* outcompeted the obligate aerobe *Brevibacillus* in dual-species pellicles through severe competition for oxygen [88].

In addition, metabolic byproducts generated by one microorganism can be toxic for the surrounding organisms; this provides the first one a competitive advantage. In the upper respiratory tract, hydrogen peroxide is a byproduct of the *Streptococcus pneumoniae* metabolism that diminish cell viability of *Neisseria meningitidis* and *Moraxella catarrhalis* [89].

Bacterial competition can also be driven by the production of small antimicrobial compounds, such as colicins, microcins, and bacteriocins. For example, *Streptococcus salivarius* in the oral cavity secretes bacteriocins that inhibit several Gram-positive pathogens, such as *Streptococcus pneumoniae* [90].

Contact-dependent growth inhibition mediated by the type 6 secretion system (T6SS) is able to inject a toxic molecule into other competitor bacteria. In this regard, T6SS of *Burkholderia thailandensis* conferred an ecological advantage to this species in mixed biofilms because it protected *B. thailandensis* from invasion by other competitor species, for example, *Pseudomonas putida* [91].

Finally, interference competition may occur by alteration of biofilm development. Bacteria can use several biofilm-inhibiting strategies including: (i) quorum sensing inhibition as a result of degradation of quorum sensing molecules or by blocking its synthesis [92,93], (ii) inhibition of adhesion by modifying the surface with biosurfactants or by down-regulating adhesion molecules [94,95], (iii) matrix degradation caused by secreted enzymes [96,97], and (iv) the induction of biofilm dispersal on the competitor species by secreting specific messenger molecules [98].

## 4. Interactions between *S. aureus* and *P. aeruginosa*

**Figure 1:**
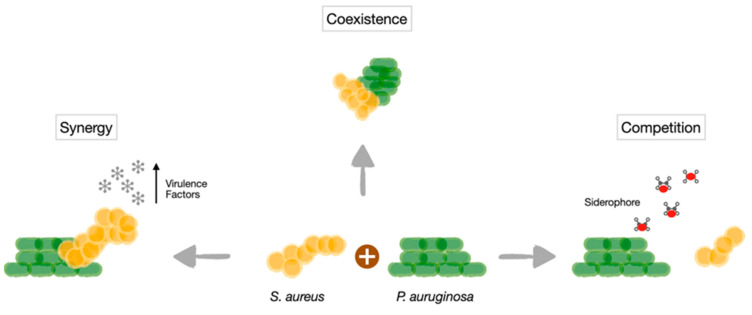
**Scheme of *S. aureus*-*P. aeruginosa* interactions.** Coexistence has been observed, with each bacterial species occupying a discrete niche. Competitive interactions mediated by secreted *P. aeruginosa* molecules, such as the siderophores pioverdine and piochelin, have also been reported. Synergistic effects with increasing production of virulence factors also occur.

*S. aureus* and *P. aeruginosa* are two common microorganisms colonising chronic wounds [50,99,100]. These two organisms, used as model organisms to study polymicrobial interactions, have been shown to display both cooperative and competitive interactions within the wound (Figure 1). The subtle balance between the competitive and cooperative behaviours of *S. aureus* and *P. aeruginosa* could be the key to understanding this interspecies relationship.

### 4.1. Interactions Observed In Vitro in Co-Cultivation Experiments

Several competitive interactions between *S. aureus* and *P. aeruginosa* have been observed by performing co-cultivation experiments under standard laboratory conditions. *P. aeruginosa* excretes several small respiratory toxins that kill or inhibit growth of *S. aureus*, including pyocyanin that permeates the cells where it produces reactive oxygen species [101,102]; the quorum sensing effector molecule 2-heptyl-4-hydroxyquinoline n-oxide (HQNO) [103]; the LasA protease (also known as staphylolysin) that cleaves the *S. aureus* peptidoglycan and induces its lysis [104]; rhamnolipids, which present antiadhesive and dispersing properties on *S. aureus* biofilms [105,106]; and the iron-chelating siderophores pyoverdine and pyochelin [107].

In response to this antagonistic attack, *S. aureus* reduces its metabolism, favouring small-colony variant selection as a survival strategy [108]. These *S. aureus* small-colony variants are well known for stable aminoglycoside resistance and persistence in chronic infections [109,110].

In vitro co-cultivation experiments using a wound-like medium demonstrated that the quorum sensing systems of *P. aeruginosa* are inhibited by the albumin present in the serum; consequently, the bacteria was unable to produce the virulence factors that kill *S. aureus* such as HQNO. This results in the survival of *S. aureus* in the presence of *P. aeruginosa* [111].

### 4.2. Interactions Observed in Wound Infection Models

In contrast to the reported antagonisms described above, the results obtained in wound infection models showed coexistence between *S. aureus* and *P. aeruginosa*. Studies during early stages of wound coinfection evidenced a predominance of *S. aureus* in non-attached bacterial aggregates and biofilm, favouring the subsequent attachment of *P. aeruginosa* to human keratinocytes [112]. Moreover, *P. aeruginosa* promoted *S. aureus* invasion to these cells. Co-infected keratinocytes showed an intermediate inflammatory response that is in agreement with the maintenance of low-level tissue damage and can be associated with chronicity, prolonged colonisation, and impaired wound repair [112].

In addition, *P. aeruginosa* showed a higher tolerance to gentamicin in *S. aureus/P.aeruginosa* polymicrobial infection when compared to mono-infection in a murine chronic wound infection model [113].On the other hand, the presence of *P. aeruginosa* induced the expression of *S. aureus* virulence factors alpha-toxin and Panton-Valentine leukocidin in a porcine wound model when compared to infection with *S. aureus* alone [50]. A recent report showed that *S. aureus* inactivated the *P. aeruginosa*-derived siderophore pyochelin via the methyltransferase Spm (staphylococcal pyochelin methyltransferase), increasing *S. aureus* survival during in vivo competition with *P. aeruginosa* in a murine wound co-infection model [114]. Furthermore, the secreted *P. aeruginosa* molecule HQNO induced the production of *S. aureus* membrane-bound pigment staphyloxanthin (STX), which consequently promotes resistance of both pathogens to innate immune effectors such as hydrogen peroxide [115].

Analysis of chronic wound biopsies suggests that *S. aureus* and *P. aeruginosa* occupy distinct niches, albeit separated by a few hundred micrometres [116]. In the same way, using a mouse chronic wound model, it has been observed that *S. aureus* and *P. aeruginosa* coexist at high cell densities in murine wounds, establishing a patchy distribution [117,118]. A precise microbial spatial distribution at both the macro (mm)- and micro (μm)-scales was mediated by *P. aeruginosa* production of the antimicrobial HQNO, while pyocyanin had no impact. This precise spatial structure enhances *S. aureus* tolerance to aminoglycoside antibiotics but not vancomycin [117]. Pougetet al. found that the percentages of biofilm formation were significantly higher in the mixed *S. aureus/P. aeruginosa* biofilm compared to those determined for the bacterial species alone and that *S. aureus* aggregates were located close to the wound surface, whereas *P. aeruginosa* was located deeper in the wound bed [118].

## 5. Interactions of *S. aureus* with Microorganisms other than *P. aeruginosa*

### 5.1. S. aureus and Enterococcus faecalis

**Figure 2:**
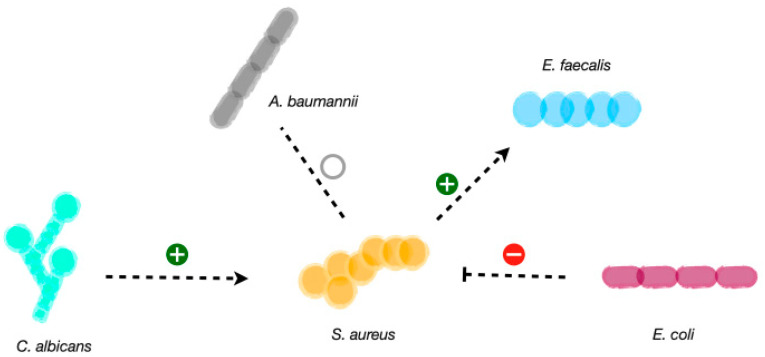
**Scheme of the studied microbial interactions of *S. aureus*.** *S. aureus* can establish neutral interactions and co-exist with *A. baumannii*. Competitive interactions have been reported for *E. coli* on *S. aureus* trough the genotoxin colibactin. Synergistic interactions occur between *S. aureus* and *E. faecalis*, where heme cross-feeding facilitates oxidative respiration in *E. faecalis*. *C. albicans* also favors *S. aureus* proliferation, biofilm formation and virulence factors upregulation.

*S. aureus* and *E. faecalis* have been implicated in biofilm-associated infections such as chronic wounds, among others [27,119]. The transfer of vancomycin resistance genes from *E. faecalis* to *S. aureus* has been observed in clinical settings [120,121]. Additionally, it has been reported that in combination, these two species act synergistically, producing augmented biofilm biomass (Figure 2) [122]. For this, heme cross-feeding has been reported, and it was postulated to involve gelatinase-mediated heme acquisition by *E. faecalis* from secreted *S. aureus* hemoproteins. Heme acquisition by *E. faecalis* facilitates its oxidative respiration [122].

### 5.2. S. aureus and Escherichia coli

*S. aureus* and *E. coli* are among the most frequent cultured microorganisms from wound infections [12,27,123]. By using a mouse excisional wound model, *E. coli* was shown to antagonize the growth of *S. aureus* via the genotoxin colibactin (Figure 2) [124]. The prevalence of polyketide synthase island (*pks*) in *E. coli* isolated from human wound swabs was nearly 30% [124]. While the mechanism for colibactin release from *E. coli* or penetration into target cells is not known, it has been shown that the colibactin intermediate N-myristoyl-D-Asn (NMDA) is able to disrupt the *S. aureus* membrane [124]. Moreover, during interspecies competition, the *E. coli* BarA-UvrY two-component system senses *S. aureus* and responds by upregulating *pks* island gene expression [124]. Given that *E. coli* and *S. aureus* are co-isolated from wounds, it may be possible that these *E. coli* strains are unable to express the *pks* island. Another possibility is related to a spatial segregation within wound biofilms such that colibactin-producing *E. coli* resides far enough from *S. aureus* to not be able to affect its viability [125].

### 5.3. S. aureus and Acinetobacter baumannii

Wound co-infections with *S. aureus* and *A. baumannii* are found in clinical settings. It has been reported that clinical strains of *S. aureus* and *A. baumannii* that were recovered from the same site of infection (diabetic foot ulcer) exhibit a state of commensalism between the two when co-cultured in vitro, without an effect of one another, whether beneficial or detrimental (Figure 2) [126]. More recently, evidence was published that *A. baumannii* can sense and respond to molecules secreted by *S. aureus*, modulating virulence responses, such as motility and biofilm formation [127]. In addition, it has been shown that the fitness requirements of *S. aureus* in vivowere dramatically changed by co-infection with *A. baumannii*, with around 50% of the essential genes needed during mono-infection converted to non-essential during co-infection [128].

### 5.4. S. aureus and Candida Albicans

The mixed species of *S. aureus* and *C. albicans* can cause skin infections. An increase in *S. aureus* proliferation and biofilm formation was observed in *S. aureus* and *C. albicans* dual-species culture [129]. According to the transcriptome analysis of the dual-species culture, virulence factors of *S. aureus* were significantly upregulated. Moreover, the beta-lactams and vancomycin-resistant genes in *S. aureus* as well as azole-resistant genes in *C. albicans* were also significantly increased [129].

### 5.5. S. aureus and Commensal Skin Bacteria

It has been demonstrated that co-infection of *S. aureus* with commensal skin flora can increase *S. aureus* virulence. This effect, termed augmentation, has been observed in several infection models, including mouse soft-tissue infection [130]. A natural mix of mammalian skin microflora, as well as isolated Staphylococcus epidermidis or Micrococcus luteus strains, was able to augment *S. aureus* virulence. Moreover, pathogenesis augmentation could be mediated by particulate cell wall peptidoglycan from a range of Gram-positive species including *Staphylococcus epidermidis*, *Curtobacterium flaccumfaciens*, and *Bacillus subtilis*, reducing the *S. aureus* infectious dose by over 1000-fold [130]. More recently, in vitro and in vivo studies have evidenced that the molecular basis for augmentation is absorption of reactive oxygen species by augmenting material (peptidoglycan), shielding *S. aureus* from macrophage-mediated killing [131].

## 6. Conclusion and Perspectives

Polymicrobial human infections are of significant concern on human health. These infections have been reported to be more tolerant to antibiotics and to cause worse clinical outcomes compared to their single-species counterparts.

*S. aureus* in polymicrobial infections constitutes a greater medical problem than *S. aureus* in single-species infections. The complex network of microbial *S. aureus* partners and their interactions has the potential, through diversity in beneficial and/or competitive crosstalk, to accelerate, delay, or worsen wound healing. Microorganisms coexisting in the same site of infection can alter growth, gene expression, invasion ability, and antimicrobial sensitivity patterns.

Further investigations are required to better understand the multi-species interactions between *S. aureus* and co-infecting organisms to design appropriate treatment strategies and to improve the management of chronic polymicrobial skin and soft-tissue infections involving *S. aureus*.

## Figures and Tables

**Table 1:** Microbial species in polymicrobial Sa-SSTIs.

Type of Infections	Co-Infecting Microorganisms	References
Diabetic foot ulcers	Gram negative bacteria *P. aeruginosa* *Acinetobacter* spp. (*Acinetobacter baumannii*) *Escherichia coli* *Enterobacter* spp. *Citrobacter* spp. *Proteus* spp. *Klebsiella* spp. Gram positive bacteria *Enterococcus* spp. (*Enterococcus faecalis*)	[10,27]
Pressure ulcers infections	Gram negative bacteria *Pseudomonas aeruginosa* *Escherichia coli* *Proteus* spp. (*Proteus mirabilis*) *Enterobacter cloacae* Gram positive bacteria *Enterococcus* spp. (*Enterococcus faecalis*)	[29,47,48]
Burn infections	Gram negative bacteria *Pseudomonas aeruginosa* Fungi *Candida albicans*	[49,50,51,52]
